# Complete Genome Sequence of *Klebsiella quasipneumoniae* subsp. *similipneumoniae* Strain ATCC 700603

**DOI:** 10.1128/genomeA.00438-16

**Published:** 2016-05-26

**Authors:** Alysha G. Elliott, Devika Ganesamoorthy, Lachlan Coin, Matthew A. Cooper, Minh Duc Cao

**Affiliations:** Institute for Molecular Bioscience, The University of Queensland, Brisbane, Australia

## Abstract

*Klebsiella quasipneumoniae* subsp. *similipneumoniae* strain ATCC 700603, formerly known as *K. pneumoniae* K6, is known for producing extended-spectrum β-lactamase (ESBL) enzymes that can hydrolyze oxyimino-β-lactams, resulting in resistance to these drugs. We herein report the complete genome of strain ATCC 700603 and show that the ESBL genes are plasmid-encoded.

## GENOME ANNOUNCEMENT

*Klebsiella pneumoniae* is a common opportunistic pathogen that can infect both plants and animals (1, 2). It is widely recognized as an urgent threat to human health because of the emergence of multidrug-resistant and hypervirulent strains associated with hospital- and community-acquired infections (3). The species was formerly classified as containing three phylogroups, namely, KpI, KpII, and KpIII (4). However, recent studies have suggested that the phylogroup KpII should be reclassified as a new species: *K. quasipneumoniae* with two subspecies, *K. quasipneumoniae* subsp. *quasipneumoniae* and *K. quasipneumoniae* subsp. *similipneumoniae*, corresponding to the two subgroups, KpII-A and KpII-B, respectively (5).

In this report, we announce the first complete genome of a clinical *K. quasipneumoniae* subsp. *similipneumoniae* isolate. The strain, formerly *K. pneumoniae* K6, was isolated from urine of a hospitalized patient in Richmond, VA, USA, in 1994 and was catalogued by ATCC under accession number ATCC 700603. Susceptibility testing of the strain confirmed resistance to ceftazidime and other oxyimino-β-lactam antibiotics (6). As routine susceptibility testing methods often fail to detect accurate extended-spectrum β-lactamase (ESBL) profiles (7, 8), this strain was selected by the National Committee for Clinical Laboratory Standards (NCCLS) as a quality-control strain to improve detection of ESBLs in *Enterobacteriaceae* (9). To the best of our knowledge, this is the first report of the complete genome of this species, in which the complete plasmid sequences encoding resistance elements are identified.

Genomic DNA was subject to whole-genome sequencing with the PacBio RS II platform on two SMRT cells with over 380-fold coverage. Sequence reads were assembled with HGAP pipeline (10) to obtain three contigs of lengths 5.31 Mb, 172 kb, and 100 kb, respectively. These contigs were circular, suggesting that they were a chromosome and two plasmids (pKQPS1 and pKQPS2). This was confirmed by submitting the sequences to the PlasmidFinder server (11). The sequences of the two plasmids were slightly longer than previous estimates of 160 kb and 80 kb (6). The GC content in the chromosome sequence was 58%, while that of both plasmids was 52%. The annotation of the genome using NCBI Prokaryotic Genome Annotation Pipeline (release 2013) contained a total of 5,394 coding genes, including 182 in plasmid pKQPS1 and 120 in plasmid pKQPS2.

The genome was submitted to the ResFinder server (12) to identify acquired antimicrobial resistance genes. While the chromosome was found to contain one resistance gene (beta-lactamase gene blaOKP-B-7), plasmid pKQPS2 harbors four resistance genes, including aadB (aminoglycoside), sul1 (sulfonamide), and the ESBL encoding genes blaOXA-2 and blaSHV-18. The presence of a chromosomally encoded OKP-B gene supported the classification of this strain as *K. quasipneumoniae* subsp. *similipneumoniae* (13). Using the MLST database, the strain was found to belong to sequence type ST498. By establishing a phylogenetic tree from the MLST genes of ST498 together with 124 other sequence types from Maatallah et al. (2014) (14), the strain was placed in the KpII-B phylogroup, further supporting the classification to the subspecies *Klebsiella quasipneumoniae* subsp. *similipneumoniae*.

### Nucleotide sequence accession numbers.

This complete genome has been deposited in DDBJ/ENA/GenBank under the accession numbers CP014696, CP014697, and CP014698.
